# Vigour of self-paced reaching movement: cost of time and individual traits

**DOI:** 10.1038/s41598-018-28979-6

**Published:** 2018-07-13

**Authors:** Bastien Berret, Carole Castanier, Simon Bastide, Thomas Deroche

**Affiliations:** 1 CIAMS, Univ. Paris-Sud, Université Paris-Saclay, F-91405 Orsay, France; 2 CIAMS, Université d’Orléans, 45067 Orléans, France; 3 Institut Universitaire de France (IUF), Paris, France

## Abstract

People usually move at a self-selected pace in everyday life. Yet, the principles underlying the formation of human movement vigour remain unclear, particularly in view of intriguing inter-individual variability. It has been hypothesized that how the brain values time may be the cornerstone of such differences, beyond biomechanics. Here, we focused on the vigour of self-paced reaching movement and assessed the stability of vigour via repeated measurements within participants. We used an optimal control methodology to identify a cost of time (CoT) function underlying each participant’s vigour, considering a model of the biomechanical cost of movement. We then tested the extent to which anthropometric or psychological traits, namely boredom proneness and impulsivity, could account for a significant part of inter-individual variance in vigour and CoT parameters. Our findings show that the vigour of reaching is largely idiosyncratic and tend to corroborate a relation between the relative steepness of the identified CoT and boredom proneness, a psychological trait relevant to one’s relationship with time in decision-making.

## Introduction

Imagine that you decide to go to your favorite restaurant. By walking fast, you could save time and have lunch earlier at the price of spending more physical effort. By walking more slowly, you could reduce effort but this would inevitably delay reward acquisition and postpone future events. If several choices with equivalent effort are available, you would certainly prefer the least time-consuming solution. Hence, time seems to be a critical determinant of daily life decisions and it may profoundly affect the control of movement vigour, that is, the interplay between amplitude, speed, duration or frequency of movements^1^. Any goal-directed movement turns out to have a certain pace whose rationale remains to be better understood despite recent developments^2–5^. Strikingly enough, people may exhibit very divergent movement speeds even for ordinary motor tasks such as saccades^6^ or pedestrian walking^7^. While biomechanics (e.g. anthropometry, energetics…) may partly account for inter-individual discrepancies, it has been shown that speed-related choices significantly depend on other factors such as reward outcome^5,8–11^. Since the brain tends to discount the value of future reward during decision-making^12,13^, researchers hypothesized that inter-individual differences in vigour may arise from the way the brain values time in the neural control of movement. This is referred to as the “cost of time” (CoT) hypothesis^6,14,15^. Time may incur a cost for a multitude of reasons in motor control^16^ but what seems clear is that humans are remarkably reluctant to move the arm slowly^17^ and that they judge long-lasting reaching movements more effortful than comparable movements of shorter durations^18^. Hence, both the biomechanical cost of movement and a cost growing with the passage of time may matter during arm movement. The origin of such a time penalization is however poorly known. Testing whether it relates to specific individual factors, be they biomechanical or psychological, could thus advance our knowledge on this issue.

**Figure 1:**
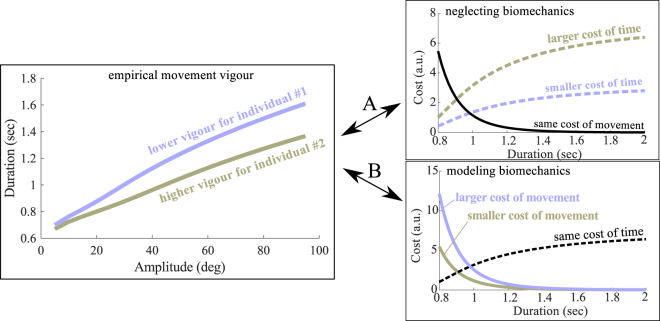
Rationale of a model-based approach. Movement vigour can be characterized empirically during reaching via amplitude-duration relationships. Assume that participant #1 exhibits a higher vigour than participant #2 (depicted in lilac and taupe, respectively; *left inset*). The utility of using a model-based approach relying on a time/effort compromise is as follows. One may be tempted to directly relate empirical vigour to some cost of time or temporal discounting of reward function, and deduce that individual #1 penalizes time more firmly than individual #2. This would be the case if one neglects anthropometric differences among individuals (*top-right inset*, taupe and lilac dotted lines; scenario (**A**). The black solid line illustrates the cost of movement for a given amplitude, which would then be the same for the two participants. However, if one takes into account the anthropometry of individuals, one may actually conclude that the two individuals possess the same CoT (*bottom-right inset*, black dotted line; scenario (**B**). This is because individual #1 may have for a larger cost of movement than individual #2 (e.g. a larger arm’s inertia), thereby affecting his/her resultant vigour. The difficulty arises from the fact that scenarios A and B would exactly lead to the same predictions of movement vigour as shown in the *left inset*, yet with contradictory interpretations regarding time penalization in both individuals. These considerations motivate the use of a model-based approach taking into account (a measure of) the biomechanical cost of movement, as in scenario B, to readjust CoT estimations.

To this aim, accurately inferring the CoT (sometimes called “reward function” by restriction) is essential, as stressed by Shadmehr and colleagues^19^: “If we could find robust techniques to measure [parameters] in the reward function of individuals, it would be possible to test for within-subject correlation between the reward function and movement kinematics”. Such a technique was recently derived to uncover the CoT underlying the vigour of an individual performing self-paced movements^4^. The methodology relies on optimal control theory, which is an appealing tool for our purpose since it inherently takes into account the biomechanical cost of movement. The model assumes a trade-off between time and effort to predict an individual’s movement vigour^2,4^. In this normative framework, vigour and CoT are *a fortiori* related quantities but their link may be intricate because of the characteristics of the moving body. Figure 1 exemplifies a situation where two subjects exhibit divergent movement vigours but nonetheless share the same CoT if their anthropometric discrepancies, which conceivably induce different biomechanical costs of movement, are taken into account. In other words, vigour is an empirical measure potentially affected by biomechanics whereas the CoT is assumed to be a higher-level function that expresses the cost entailed by the passage of time during goal-directed movement.

The purpose of the present study was to investigate the vigour of self-paced arm reaching movements, with a focus on inter-individual variability to clarify the relation between the time cost inferred from such optimal control techniques and certain individual factors. Here, we considered a simple arm pointing task in the horizontal plane, with a fully extended arm involving only rotations about the shoulder joint in order to constrain hand paths and, therefore, emphasize speed-related choices. We quantified the vigour exhibited by 38 participants across repeated measurements within/between sessions. A low intra-individual variability of vigour would indicate that it represents a relatively stable characteristic of an individual for the task under investigation, which could be accounted for by individual traits. In that case, time costs that replicate the vigour of each individual would be inferred using a model of the biomechanical cost of movement. The link between anthropometric traits related to limb biomechanics (e.g. arm’s inertia) or personality traits related to decision-making (e.g. impulsivity^6,20^ or boredom proneness^21^) and CoT parameters could then be explored. Alternatively, a large intra-individual variability would indicate that vigour strongly varies across repeated measurements. Hence, these fluctuations would be hardly predictable by stable individual traits as those related to inertia and one’s relationship with time.

## Results

### Inter-individual versus intra-individual variability of movement vigour

**Figure 2:**
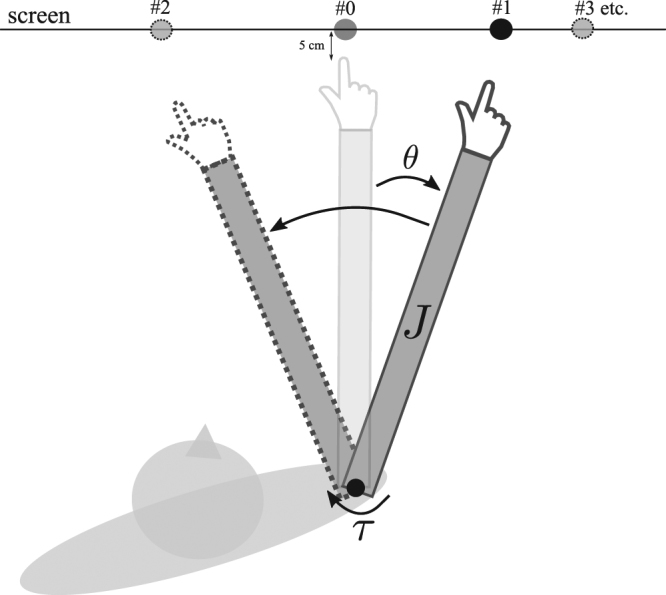
Illustration of the task. Top view of a participant performing a series of arm pointing movements in the horizontal plane. A block was composed of 20 movements and always started with the arm positioned perpendicularly to the screen and aligned with its center (transparent arm). A randomized sequence of 20 targets was then triggered by ensuring 2 movements for each of the 10 tested amplitudes (from 5° to 95° with a 10°-step). In this example, a first target was turned on (#1) and the subject had to point to it and stay still (plain arm) until the next target (#2) was turned on (target #1 was simultaneously turned off). The participants had to stay still (dotted arm) until the process was continued (target #3 and so on). At the end of the block the participant was asked to relax the arm for at least 2 minutes. Importantly, the total duration of a block was fixed across participants (a block lasted 75 seconds). Therefore, whatever a participant tended to move fast or slow, the duration of the experiment was the same and the time spent to compensate for gravity was fixed across participants, thereby preventing an effect of a gravity-related cost in inter-individual differences of vigour.

**Figure 3:**
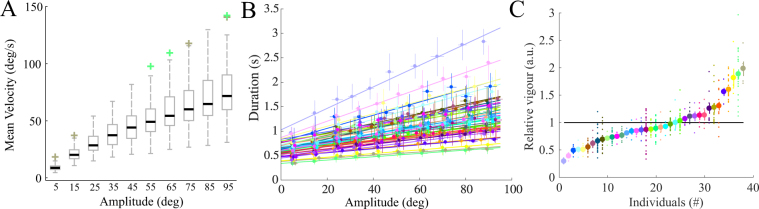
Inter- and intra-individual differences in movement vigour. (**A**) Boxplots of the mean velocities across individuals for each stimulus amplitude (ranging from 5° to 95°) during the first session. Rectangular boxes represent the 25th and 75th percentiles, thick black lines are medians, whiskers cover approximately 99.3% of the data. Crosses depict potential outliers. (**B**) Amplitude-duration relationships for the 38 participants exhibited when all data were pooled together (sessions, blocks) and the 10 amplitude stimuli were clustered. Each color stands for one participant (color code is kept fixed throughout the paper). For each participant, all the trials of the first session corresponding to each stimulus were averaged (error bars are standard deviations). Affine fits were applied separately for each participant, from which vigour was defined as the inverse of its slope. (**C**) Relative vigour evaluated for each individual. Individuals were ordered by ascending score of average vigour (large dots). Small dots are repeated measurements obtained for each block of every available session for each participant (n = 425). The horizontal black line indicates the mean vigour of our population.

Participants were asked to move at a comfortable pace such that they selected the vigour of their movements by themselves (see Fig. 2 and Methods for more details about the task). In each session, 5 blocks of 20 distinct arm pointing movements were recorded. Each block lasted 75 seconds such that the total duration of the experiment was constant across participants. Ten amplitudes, ranging from 5° to 95°, were tested by varying the location of quite large target disks (3 cm diameter) displayed on a big projection screen. Importantly, participants were not asked to move as fast as possible nor to be as accurate as possible, which contrasts with traditional speed-accuracy trade-offs studies^22^. In these self-paced reaching movements, the 38 participants exhibited a grand mean speed of 46.33 ± 15.11 deg/s (mean ± std across subjects, all amplitudes pooled together) [ranging from 20.14 to 84.13 deg/s]. Inter-individual differences in terms of mean velocities are reported as a function of the stimulus amplitude in Fig. 3A (boxplots). For all tested amplitudes, noticeable inter-individual differences existed in terms of preferred overall velocities even though the present reaching task was relatively basic. In general, speed varied by about 50% from the 25th and 75th percentiles of the population (a similar value to what was observed in^6^ for saccades) and by about 200% between the two whiskers (whiskers covered 99.3% of the approximately normally distributed data). We then estimated a movement vigour score for each participant based on the change of movement duration as a function of amplitude. We found that the relationships between amplitude and duration were well approximated by affine fits for the present self-paced reaching movements, in agreement with previous studies^4,22^. In Fig. 3B, the empirical amplitude-duration relationships are depicted for every participant (pooling repeated measures and clustering amplitudes; *R*^2^ = 0.96 ± 0.03, *T* = (0.008 ± 0.004) × *A* + (0.63 ± 0.15), *p* < 0.001 where *T* and *A* are motion duration in seconds and amplitude in degrees, respectively). Again, remarkable inter-individual differences are noticeable on the slopes (and to a lesser extent intercepts). In these data, slopes and intercepts positively correlated (*R* = 0.81), meaning that, in general, participants with a large slope also tended to exhibit a large intercept. Interestingly, common to all participants was the tendency to increase motion duration with its extent. In the present work, movement vigour was defined as the inverse of the slope of this affine relationship (see Methods). A relative vigour score (normalized by the average vigour found in our population as in^6^) was then computed and the results are presented in Fig. 3C. Participants were sorted by increasing average vigour. In this panel, vigour was computed for all available repeated measurements of each participant, that is, for each of the 5 blocks of each available session. This was done to decompose inter-individual variability versus intra-individual variability. Some participants were 2 to 4 times more vigorous than others on average. Yet, some intra-individual variability was also clearly visible. In order to quantify the stability of vigour within and between sessions compared to inter-individual differences, we performed a statistical decomposition of variance using a hierarchical linear model (HLM, see Eq. 8 in Methods). Results are reported in Table 1. Remarkably, most of the variance in movement vigour was rooted in inter-individual differences rather than intra-individual differences. The estimated respective proportions of variance in vigour scores were 72.0% versus 28.0% (with respect to the total variance).

**Table 1:** Statistical decomposition of variance of relative vigour scores.

	Level 1 variance [Ω_*e*_] (between blocks)	Level 2 variance [Ω_*v*_] (between sessions)	Level 3 variance [Ω_*u*_] (between individuals)
Proportion of variance	17.0% [12.4;22.8]	11.0% [10.0;11.8]	72.0% [67.2;75.8]

We used hierarchical linear models (empty model, Eq. 8) to obtain a statistical decomposition of variance. The estimated percentages of variance (with respect to the total variance) across blocks (level 1), sessions (level 2) and individuals (level 3) respectively, using all intra- and inter-individual observations (n = 425), are reported. Confidence intervals are provided in brackets.

In the following, we thus focus on inter-individual differences and investigate whether certain traits can account for a significant portion of these relatively stable inter-individual differences of vigour.

### Relation between empirical vigour scores and individual traits

**Figure 4:**
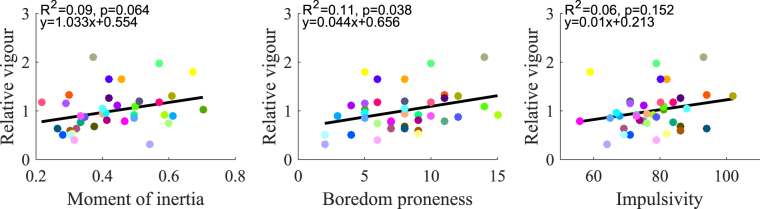
Regression analyses between empirical vigour scores and individual traits. Linear regressions between moment of inertia (left), boredom proneness (middle), impulsivity (right) and relative vigour. Only a significant positive trend was observed for boredom proneness (p = 0.038).

We first analyzed whether inter-individual variations of movement vigour could be accounted for by anthropometric or psychological traits (Fig. 4). Linear regression analyses were performed. Although the relationships between vigour and inertia, boredom or impulsivity were always positive, only a significant trend was found between boredom proneness and vigour (p = 0.038). Boredom proneness accounted for 11% of the inter-individual variance of vigour. Note that in our sample, impulsivity and boredom proneness scores positively correlated across participants with *R* = 0.55. Hence, although these two personality scores are linked, one variable could only explain 30% of the variance of the other. This justifies that we performed separate analyses for these two psychological traits.

As stressed in the Introduction, empirical vigour scores are potentially affected by the actual biomechanical cost of movement, which may differ between individuals at least because of anthropometry. To refine these results and test the extent to which inter-individual differences of vigour may relate to inter-individual differences in a valuation of time, a model-based approach was conducted. In particular, one objective was to check whether or not certain CoT parameters could be linked to the relationship with time of individuals.

### Identification of individual time costs

We relied on the theory of optimal control for arm reaching movement^23^. More specifically, we considered the CoT theory that assumes that any movement entails a cost which is composed of a biomechanical effort term and a time cost term^4,24^. Acknowledging that movement normally requires spending both time and effort, it hypothesizes that the planning of movement time results from the minimization of an effort/time compromise.

**Figure 5:**
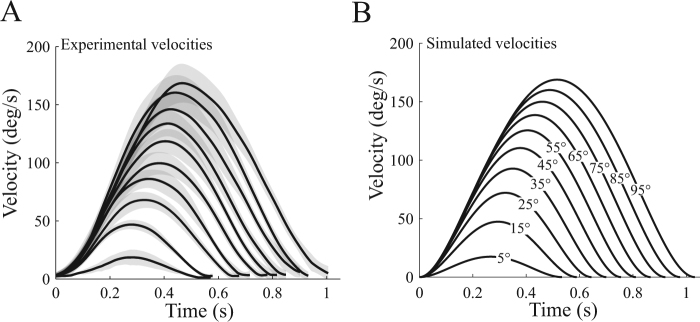
Example of velocity profiles for an individual. (**A**) Experimental velocity profiles for each amplitude for an individual with relative vigour of 1.23, within the first session. For each amplitude, velocities were averaged across 10 repetitions (black solid lines) and shaded areas correspond to standard deviations. (**B**) Corresponding simulated velocity profiles as predicted by the (free-time) optimal control model. Note that both the movement time and the shape of velocity profiles was predicted by the model (after a correct cost of time had been identified).

Here the model of the biomechanical cost of movement was based on the integral of squared joint torque, as follows:

1 $$L(T)={\int }_{0}^{T}[{\tau }^{2}+\varepsilon {u}^{2}]dt,$$

where *τ* is joint torque and *u* is angular jerk (see Methods for details). The rationale is that it is in agreement with recent findings suggesting that what makes a reach movement effortful may scale quadratically with force^18^. However, squared torque alone does not allow to predict biological velocity profiles in an optimal control model because peak velocities tend to be under-estimated. The cost was thus regularized by a quadratic control cost (term *εu*^2^ in Eq. 1). The free parameter *ε* was fixed to 0.005 throughout the paper but we verified that varying this parameter between 0.0001 and 0.1 did not affect our conclusions. Tuning this parameter has actually an effect on simulated peak velocities. A common index used in arm movement studies is the peak to average velocity ratio. We chose the value of *ε* such that this ratio was about 1.87 in simulation because it is considered as a normal biological value (e.g.^25^). In our experiment, the experimental mean peak to average velocity ratio was 1.86 ± 0.04 across subjects. In Supplementary Information, we also consider a measure of the biomechanical cost of movement based on squared torque change^26^, which has no free parameter like *ε*, and which also produces realistic bell-shaped velocity profiles. For the sake of completeness, we tested a cost of movement based on the angle jerk alone as it also predicts realistic velocity profiles (term in *u*^2^ in Eq. 1). The latter would however represent a measure of effort independent of limb’s inertia. As such, it can be viewed as a “subjective” measure of effort in the sense that the cost of the same movement would be identical across participants. An illustration of the experimental velocity profiles is given in Fig. 5A. The proposed model and cost function predicted velocity profiles that were similar to experimental ones, as depicted in Fig. 5B. We stress that it was a prerequisite for us to have a model of the biomechanical cost of movement that at least predicts realistic arm trajectories when movement time *T* is preset by the modeler; it would indeed make no sense to correctly predict movement time if the associated arm trajectory is unrealistic. It is worth noting that in the reported simulations (Fig. 5B), we did not preset movement times by hand; our model predicted the correct experimental movement times for each amplitude thanks to a well-identified CoT. Before introducing the CoT, we note that the cost *L*(*T*) estimates the biomechanical cost necessary to drive the arm from an initial resting position to a final resting position, in time *T*. This cost implicitly depends on the arm dynamics and motion amplitude, and explicitly depends on movement duration *T*. The arm dynamics was modeled as $\tau =I\ddot{{\theta }}+b\dot{{\theta }}$ where *θ* is the shoulder joint angle, *b* is a constant friction coefficient and *I* is the arm’s moment of inertia whose value was adjusted to each participant using documented anthropometric tables^27^. Although *b* may also vary across individuals, it is rather involved to estimate it accurately experimentally (see^28^ for instance). Here we simply tested the sensitivity of our results by varying *b* between 0 (neglecting frictions) and 1 (about maximal values estimated in^29^) and we found that it had very little influence on our results.

In the present optimal control model assuming an effort/time trade-off, we further postulate that a CoT adds to *L*(*T*) such that the total cost to minimize is *C*(*T*) = *L*(*T*) + *G*(*T*) with:

2 $$G(T)={\int }_{0}^{T}g(t)dt,$$

where *g* is an unknown (non-negative) time-varying function. The function *G* is called CoT. Here we assume that *G*(0) by convention.

**Figure 6:**
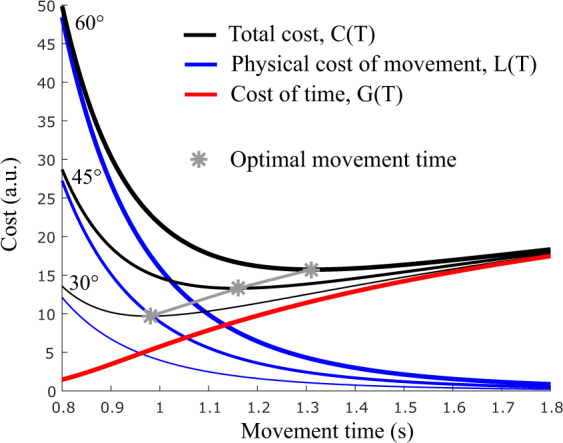
Illustration of the time-effort compromise model. The biomechanical cost of movement is depicted in blue and it depends on the motion amplitude. In classical motor control models, it is a decreasing function of time. The cost of time is depicted in red. Note that it is independent of motion amplitude and it is growing with time. The total cost, sum of these two latter costs, yields optimal movement times (emphasized by gray stars). These predicted durations will match experimental ones for a suitable choice of the time cost. Uncovering the correct time penalization is the goal of the CoT identification procedure,

Concretely, once a biomechanical cost of movement is assumed, the CoT will exactly yield the missing part of the total cost required to predict the correct movement times for each tested amplitude. If the cost of movement alone predicts the correct durations, then the inferred CoT will be null. If the cost of movement alone predicts slower movements than actually produced by the participants, then a positive CoT will be necessary to explain why movements are not slower. It is worth stressing that, by definition, the CoT function *G* does not depend on the limb’s trajectory, anthropometry or motion amplitude. It just penalizes movement time according to some function *G* that is for the moment unknown/unidentified. Mathematically, the CoT is modeled as a pure time cost (*G* is function of movement time *T* only) but in practice it can capture any phenomenon that behaves like a CoT without being a genuine time cost in the sense of an explicit processing of time. As such, the proposed model can account for any cost that grows with time independently of the actual arm trajectory (see Discussion). Figure 6 illustrates the present effort/time optimal control model.

Using a free-time optimal control model of motor planning, we showed in previous studies that the value *g*(*T*) can be computed accurately from the resolution of the associated optimal control problem in fixed time *T* minimizing the biomechanical cost of movement, *L*(*T*), alone (see^4,30^). From an experimental movement of duration *T*, one can thus infer the value *g*(*T*). However, to recover *G*, we need to integrate *g* and therefore different values *g*(*T*) are required. This is possible in practice if we are able to record self-paced movements of different durations. The results shown in Fig. 3B reveal that this is possible by varying the amplitude of the stimuli. From the empirical vigour exhibited by each participant, we were able to sample *g*(*T*) values for times *T* corresponding to movement times estimated for each amplitude. By visual inspection, we noticed that *g* exhibited an inversion of its rate of change on the time interval where it could be inferred from experimental data, thereby suggesting a sigmoidal nature for the integrated CoT *G*. This is in agreement with^4^ where the robustness of this sigmoidal shape was tested in depth by varying modeling of the arm’s dynamics, measure of the cost of movement etc. On this basis, we decided *a posteriori* to fit *G* with a sigmoidal function of the form

3 $$G(T)=\alpha -\frac{\alpha }{1+{(\frac{T}{\delta })}^{\beta }}.$$

In this model, *α* is the upper asymptote of *G*, *β* is the relative steepness of the curve, and *δ* is the inflection point location (it is the time such that $G(\delta )=\frac{\alpha }{2}$). In Supplementary Information, we also fitted the CoT to asymmetrical sigmoidal functions of the form $G(T)=\alpha -\alpha /{(1+{(\frac{T}{\delta })}^{\beta })}^{0.1}$ because visual inspection suggested that the growth of *g* could actually be larger than its subsequent decrease. This CoT may be compared to previous functions used to model the temporal discounting of reward, in particular when a hyperbolic model was assumed in which case we would have $G(T)=\alpha -\frac{\alpha }{1+\beta T}$ where *α* and *β* were respectively referred to as “reward” and “impulsivity”^2^. In our framework, *α* is not necessarily restricted to the nature of a reward (though the inferred CoT should include it). It is just the limit of the cost of time for extremely slow movements. Note that it is actually unclear whether or not *g* decreases to zero for infinite time, but for simplicity and comparison with the existing literature, we assumed it was so in this study. Parameter *β* expresses how rapidly time is penalized, which may thus be a parameter that relates to one’s relationship with time (e.g. impulsivity). Parameter *δ* conveys information about when time is maximally penalized by an individual. Usually, temporal discounting functions do not have such a parameter but here it was required to shift the sigmoid to the correct location on the time axis and get reasonable goodness of fit.

**Figure 7:**
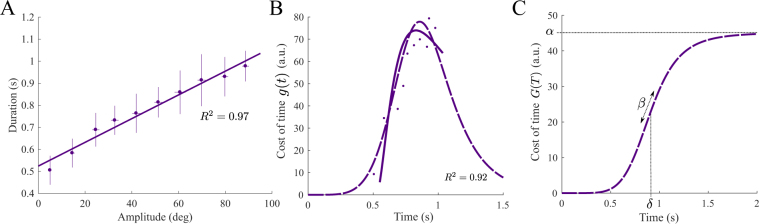
Identification and fitting of the cost of time. (**A**) Amplitude-duration relationship from which the CoT values are estimated, for the individual whose velocity profiles were depicted. (**B**). The infinitesimal CoT values *g*(*t*) sampled from the fit of the data in panel A are displayed in solid line, on the time interval of empirical movement durations (y-axis in panel A). Single dots correspond to raw data points in panel A). In dashed lines, the fitted infinitesimal time costs, *g*, for the based upon the formula of the time derivative of a sigmoid function, according to Eq. 7. (**C**) Corresponding integral costs of time, *G*, obtained by replacing the best-fitting parameters in Eq. 3. The inferred CoT functions are depicted on the time intervals [0 s, 1.5 s] and [0 s, 2 s] respectively to visualization purpose. In this example, estimated CoT parameters with confidence intervals were *α* = 45.11 (41.44, 48.78) for the asymptotic value, *β* = 6.09 (5.57, 6.61) for the relative slope and *δ* = 0.91 (0.89, 0.93) for the time to peak slope.

We then identified one CoT per participant given that vigour mostly varied across participants rather than within participants. We used the motion data depicted in Fig. 3B to infer a CoT for each participant separately. A summary and illustration of the fitting procedure is given in Fig. 7 for a participant. The goodness of fit of the inferred function *g* was *R*^2^ = 0.89 ± 0.05 across participants. The rationale for the fitting was to estimate relevant parameters of the CoT function in an automated way and to subsequently try to relate them to certain individual traits. The best-fitting parameters were as follows: *α* = 43.7 ± 71.2, *β* = 5.52 ± 1.05, and *δ* = 1.19 ± 0.39 (mean and std across participants). We checked whether there were some co-variations among the identified CoT parameters. For instance, linear regression analysis between log *α* and *β* revealed that 49% of the variability between these two parameters could be accounted for. The log transformation appeared to be relevant to perform linear analyses with *α*. Respectively 80% and 46% of the variability could be explained for *δ* vs log*α* and *δ* vs *β*. These analyses showed that *β* may capture specific inter-individual variations that are not captured by either log*α* or *δ*.

It can be expected from the modeling that the inferred time costs must relate in some way to empirical vigour scores given the identification process. However, this identification relied on an estimate of the biomechanical cost of movement for each participant and, therefore, non-trivial relationships may arise from the optimal control computations. Linear regressions were performed to clarify the relationships between vigour and estimated CoT parameters. It appeared that log*α* explained 89% of the inter-individual variability of vigour (p < 0.001). Regarding *β*, it explained 68% of vigour variability. Parameter *δ* explained 69% of the variability in vigour but it was in fact more strongly related to the intercepts of the fitted amplitude-duration relationships (91% of variance accounted for). In this work, we focused on a definition of vigour based on slopes, rather than intercepts, of these relationships (see Methods).

### Relation between CoT parameters and individual traits

**Figure 8:**
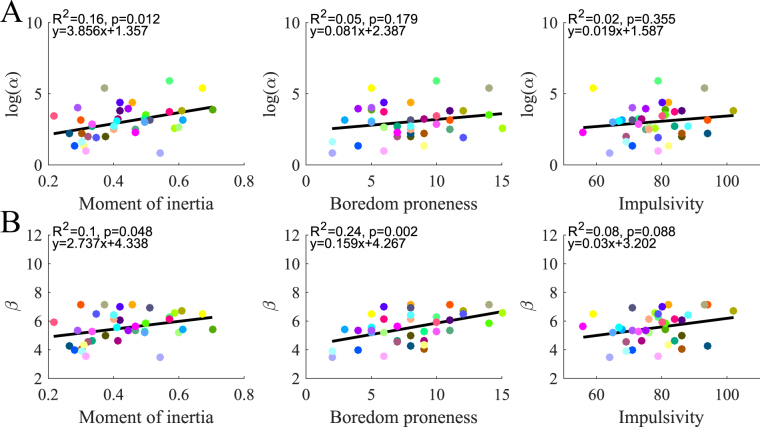
Regression analyses between the main CoT parameters and individual traits. (**A**) Relationships between moment of inertia, boredom proneness, impulsivity and log*α*. (**B**) Relationships between moment of inertia, boredom proneness, impulsivity and *β*.

We next performed regression analyses between the CoT parameters and the individual traits under consideration (Fig. 8). Regarding logα, we tested whether part of its (inter-individual) variance could be explained by predictors such as moment of inertia, boredom proneness and impulsivity (Fig. 8A). We found a significant positive relationship between log*α* and inertia with 16% of the variance accounted for (*p* = 0.012). This relationship was robust with respect to changes of CoT fitting and dynamic effort cost (see Supplementary Information). However, when the effort cost was based on angle jerk alone, the relationship between log*α* and inertia disappeared (*R*^2^ = 0.07, *p* = 0.12). No significant explanation of variance was found between log*α* and personality traits (5%, p = 0.18 and 2%, p = 0.35, for boredom proneness and impulsivity respectively).

Regarding *β*, we found a barely significant positive relationship between *β* and inertia with 10% of the inter-individual variance being accounted for (*p* = 0.048) (Fig. 8B). In contrast, we found a significant positive relationship between *β* and boredom proneness with 24% of the inter-individual variance accounted for (*p* = 0.002). This relationship was robust with respect to the measure of the biomechanical cost of movement (squared torque change instead of squared jerk/torque) and to the choice of the sigmoidal fitting (asymmetric or asymmetric) (see Supplementary Information). When assuming an effort cost based on angle jerk alone, the relationship between *β* and boredom proneness also remained significant (*R*^2^ = 0.20, *p* = 0.005). In contrast, no significant relationship was found for impulsivity in general although a slight positive trend was observed. For the sake of completeness, we also performed regressions analyses for parameter *δ* but no significant relationship was found (not depicted).

In summary, participants with a larger moment of inertia tended to have a greater cost of time via parameter log*α*, but only for those measures of effort that scaled with inertia. Additionally, participants with larger boredom proneness scores tended to penalize time more steeply via parameter *β*, which also tended to increase their vigour.

## Discussion

In this study, we examined the vigour of self-paced reaching movements from the perspective of a cost of time (CoT) theory within the optimal control framework. We considered a simple pointing task with one degree-of-freedom to emphasize speed-related choices of 38 individuals across repeated measurements. We found that vigour was a relatively stable characteristic of an individual in this task, as revealed by decomposition of inter-individual versus intra-individual variance. We then focused on inter-individual variability and found that more prone-to-boredom participants tended to have a larger vigour. To refine these results, we identified a CoT underlying the vigour of each individual using a model-based approach that takes into account anthropometry and an estimate of the biomechanical cost of movement (see also^4,30^). Assuming the latter cost was based on squared torque/jerk or torque change, this procedure revealed that the CoT could be a sigmoidal function for all participants, the parameters of which were estimated. We then examined whether anthropometric or psychological traits could explain a significant portion of the inter-individual variance in CoT parameters. Inertia was found to be positively related to the overall amplitude of the identified CoT identified when an “objective” measure of effort increasing with arm inertia was assumed (torque, torque change); considering a more “subjective” measure of effort (jerk) canceled this effect. Boredom proneness was found to be positively linked to its relative steepness for all the effort costs investigated. These findings suggest that personality traits related to one’s relationship with time are among the factors that influence the time cost identified by our procedure. Below we discuss the scope of these findings focusing on the inter-individual differences of movement vigour, on the potential origin of the inferred CoT, and on the limitations of the study.

In a perspective paper, Kanai and Rees^31^ noted that whereas inter-individual differences have been the main focus in some areas of psychology such as personality research, this “potentially powerful approach has been almost completely neglected for many years in studies of the neural basis of more basic cognitive functions, such as perception and motor control. These studies have mostly focused on commonalities across individuals and thus often neglect inter-individual differences”. The present motor control study follows Kanai and Rees’s precept by focusing on inter-individual peculiarities. Our results revealed considerable differences of vigour across participants even for what may appear as a common/trivial motor task. Where Choi and colleagues found that saccadic vigour could increase by ∼50% from one subject to another^6^, we found in the present arm reaching task that vigour increased by ∼70% from the 25th to the 75th percentiles or even considerably more when comparing more extreme individuals. Despite marked inter-individual differences, the commonality of all participants was the tendency to increase both speed and duration as a function of amplitude. This increase of speed with amplitude seems to agree with the isochrony principle^32^ but the speed increment was insufficient to keep duration constant for these quite large movements. The increase of both speed and duration with movement extent is also verified for saccades^6^ (up to a certain point) and walking with a start and a stop^33^. Therefore, it seems to be a general and interesting signature of self-paced discrete motor tasks.

Several factors may influence the relations between distance, speed or time, that is, one’s movement vigour. Notably, anthropometry may play a key role and account for certain inter-individual differences in vigour. For locomotion, the length of the legs may conceivably affect the speed of walking as revealed by theoretical considerations based upon a simple pendulum model^34^. Yet relatively weak (though positive) correlations have been found between preferred walking speed and individual’s height^35^. For the sake of comparison, simple linear regression analyses were performed here between movement vigour and arm’s length or body mass (N = 38); they did not yield any significant result although the trend was always positive (*R*^2^ = 0.05, *p* = 0.14 and *R*^2^ = 0.07, *p* = 0.11, respectively). Hence, basic anthropometric factors did not explain a significant part of inter-individual variations of movement vigour in the present arm pointing movements. Arguably, a more meaningful variable encompassing those parameters and other physiological characteristics may be metabolic energy. An individual’s preferred movement speed may be selected such as to minimize metabolic energy expenditure. In agreement with this premise, the metabolic cost of both walking and reaching was shown to exhibit a U-shape as a function of overall speed, whose global minimum was closely related to the actually selected motion speed^2,36,37^. Nevertheless, departure from energetic optima have been observed for locomotor tasks with additional constraints or in different contexts indicating the need to consider other determinants of movement pace^38–40^. Recently, metabolic energy has been proposed as an objective measure of the biomechanical cost of movement^2^ and departure from metabolic optima was interpreted as the consequence of a superimposed temporal discounting of reward. The authors concluded that the energetic cost itself should be discounted in time to reproduce several experimental findings^2^. However, if humans temporally discount physical effort, this would tend to flatten out the second branch of the “U” curve, thereby preventing physical effort minimization alone from being able to predict an individual’s movement vigour. Indeed, discounted perceived physical effort tends to decrease monotonically in time (see effort curve in Fig. 1 in ref.^2^ or present Fig. 6) and therefore, without a CoT, very slow movements should be judged less effortful and thus preferred by participants. On the contrary, long-duration movements are judged more effortful than comparable short-duration movements^18^. In addition, humans tends to avoid overslow movements^17^. The CoT hypothesis may help to reconcile these findings if it is viewed as the temporal counterpart of physical effort which accounts not only for a temporal discounting of reward but also for any other cost that would accumulate over time and behave like a time cost. Gravity could underlie such a cost that accumulates over time and leads to the planning of faster movements; objectively, the latter could also scale with arm inertia. Yet, in seeming support of a reward discounting aspect, empirical vigour scores tended to positively correlate with boredom proneness, a personality trait linked to time-related decision making.

In an attempt to seek for more direct explanations of vigour in terms of a time cost, a model-based approach was employed and a CoT was identified for every individual using optimal control models. On the one hand, anthropometry was found to have a significant influence on *α* values (upper asymptotic value of the sigmoidal CoT), where individuals with a larger moment of inertia tended to have a larger CoT magnitude. Actually, this effect only hold when our measures of effort scaled with inertia (i.e. torque and torque change costs increase with inertia). Since our model assumes an additive time/effort trade-off, the overall CoT magnitude may naturally have to scale with the magnitude of the biomechanical cost of movement. This point was verified by assuming the squared angle jerk as an alternative measure of effort (hence ignoring arm’s inertia). Such an effort cost can be thought as a normalized (subjective) measure of effort since it postulates that the cost of movement would only depend on kinematics. Therefore, the link between inertia and the CoT magnitude seems to be mainly a by-product of modeling and our premise to focus on measures of effort that depend on arm inertia. The effect could also partly arise here because increasing inertia in the current experiment could actually lead to shorter movement times due to the accumulating effort cost related to holding the arm horizontally; this energy expenditure via heat energy loss is rarely modeled in motor control but could be implicitly captured by the identified CoT. It is noteworthy that increasing inertia at fixed CoT should actually slow down movement in our framework (as in^2^) but participants with a larger inertia did not move less vigorously in this task, which may explain why the CoT magnitude was found to increase for dynamic effort costs. On the other hand, and perhaps more interestingly, boredom proneness was found to account for about one-fourth to one-fifth of the inter-individual variance of *β* values (the relative steepness of the CoT function) depending on the chosen measure of effort. This reaffirmed what was glimpsed on empirical vigour scores. Positive yet non-significant trends were found for impulsivity as in^6^. The personality traits we tested here, related to one’s relationship with time, reveal an overall daily-life behaviour that may be exemplified in certain decision-making situations such as line queuing at the supermarket^41^ or changing lanes in traffic jam^42^. Our results suggest that the steeper time penalization of prone-to-boredom people would also manifest itself during self-paced arm movements. However, although significant results were obtained for boredom proneness, about 75% of the inter-individual variance remained unaccounted for. It is plausible that other psychological dispositions, not tested here, could explain an additional part of inter-individual variance. Otherwise, in-lab psychophysical experiments capturing the essence of daily tasks related to time/speed choices could help clarifying the link between the steepness of the movement-related CoT investigated here and a CoT underlying non-motoric decision-making processes, by providing more objective measures. Besides anthropometric and psychological traits, it is worth stressing that other individual characteristics could also play a key role such as muscular/physiological ones for which large and stable inter-individual differences have been reported (e.g.^43^) but this would remain to be investigated.

While we tested the existence of a link between the present CoT and one’s relationship with time in decision-making, we stressed that passage of time may be (or appear to be) costly for other reasons in arm movement control. For instance, as already pointed out, gravity-induced heat energy loss may entail a cost that grows linearly with time during movement, thereby bringing participants with large inertia to move faster. Although our data indicated that it was not the case in the present task (which was designed in such a way that, overall, there could be no energy saving by doing so), we cannot be exclude that gravity did not influence at all the vigour of our participants. However, it has been reported that after one month in weightlessness cosmonauts do not drastically reduce the speed of their reaching movements^44^. This suggests that even in the absence of gravity there still exists a cost that prevents reaching movements from being too slow. Alternatively, on-line sensorimotor processes could incur a cost that may be modeled adequately as a time cost independent of limb’s trajectory. Because of motor noise notably (constant but also signal-dependent), feedback corrections are necessary to correct for errors during movement execution^45^. Errors would otherwise accumulate over time and lead to potentially large endpoint inaccuracy. It is worth stressing that moving very slowly also seems hard for humans with the presence of overlapping movements^46^. Therefore, moving slowly may monopolize more neural and/or attentional resources, the total load of which would grow with time. The current CoT may capture those costs that do not relate to an explicit processing of time but may be well modeled as a time cost. Future work is however needed to disentangle the different reasons that prevent the planning of overslow movements by the CNS (i.e. a “pure” time cost versus a cost of a different nature but that can be represented as such). Thus, the CoT identified by our procedure is likely multifaceted and integrates physiological, psychological, attentional or neural components which cannot be isolated yet. Strictly speaking, the time cost we inferred in this paper is just the penalization that must be put on time to replicate empirical motion vigour, given a presumed measure of the biomechanical cost of movement.

Although we verified that our main conclusions were robust with respect to certain modeling and parameter choices, the study relies on a number of assumptions and hence presents certain limitations. First, we used a definition of movement vigour based on the steepness of the amplitude-duration relationships (which itself required estimation of movement times). Other definitions of vigour could have been used such as the one proposed in^6^, which is based on peak velocities. We however verified that the latter definition was coherent with our definition of vigour. Second, we wanted to assess whether the estimated time costs could provide original insights regarding inter-individual differences of vigour and, in particular, be linked to personality traits related to decision-making. To this aim, we had to map movement amplitudes to durations for each individual and to use an estimate of the biomechanical cost of movement. Our choices were made based on the existing literature and state-of-the-art knowledge for arm pointing movements but we cannot discard that other modeling choices could not yield different time costs estimates. Above all, this work emphasizes that an extended multidisciplinary theory-driven approach may be useful to better decipher where the idiosyncrasy of movement vigour derives from.

## Materials and Methods

### Experimental data set

#### Experimental task

Participants: Thirty-eight healthy young adults without known neuromuscular disease and with normal or corrected-to-normal vision participated in the experiment (17 females, 27.7 ± 5.1 years old, 68.7 ± 12.7 kg, 72.1 ± 4.8 cm of fully-extended arm’s length; mean ± std values). The experimental protocol was approved by the Univ. Paris-Sud EA 4532 local Ethics Committee and conformed to relevant guidelines and regulations. Written informed consent was obtained from each participant in the study as required by the Helsinki declaration. All the participants were naive to the purpose of the experiment.

Motor task: Participants performed visually-guided single-joint arm movements in the horizontal plane with their arm fully extended (rotation around the shoulder joint with their dominant arm). The same protocol was described in^4^. Briefly, participants stood in front of a large vertical screen where spotlight target disks (3 cm diameter) were displayed. Ten amplitudes ranging from 5° to 95° were tested and 10 movement repetitions per amplitude were recorded (5 in the rightward and 5 in the leftward directions). The time interval between the appearance of two successive stimuli ranged from 3.6 s for an amplitude of 5° to 4.2 seconds for an amplitude of 95°. This timing was used to take into account the increase of motion duration with amplitude. It was kept fixed for all participants such that the total duration of the experiment was the same for all participants. The time separating two stimuli was large enough to let the participants accomplish the movements at their preferred speed. Each experimental session was divided in 5 blocks of 20 trials each, a block lasting about 75 seconds. The duration of a block was thus constant across participants but they were not explicitly informed it was so. An initial familiarization phase consisting of 20 trials was performed before the recording of the 100 trials. Participants were allowed to rest and relax their arm between each block for several minutes (a “pause” word appeared on the screen to that end), in order to reduce the effect of fatigue. The sequence of amplitudes was fully randomized within a session with the constraint that 2 movements for each amplitude were included in a block (e.g. a leftward movement of x° was paired with a rightward movement of x° within a block). Among the 38 subjects, 17 were asked to perform again the task on separate days (7 subjects participated in 5 sessions, 1 subject in 4 sessions, 7 subjects in 3 sessions and 2 subjects in 2 sessions). Considering all the blocks/sessions for all the participants, we gathered a total of 8500 single movements to analyze, and a total of 425 measurements from which vigour and the cost of time could be estimated (including inter- and intra-individual variability).

Instructions: Participants were instructed to move at a spontaneous, comfortable speed^4^. The question of the very existence of a preferred speed had been investigated in a previous study^47^. Here subjects were also asked to perform one-shot smooth movements without terminal correction. Endpoint accuracy was of minor concern in this task because subjects only had to point in the direction of the target without touching it and the movements were unidimensional, visually-guided and self-paced. It is worthwhile to stress that the present study does not deal with the classical speed-accuracy trade-off (Fitts’s law), which has been the subject of extensive works (including within the cost of time theory, e.g.^30^). Instead, it focuses on self-selected/natural movement pace where Fitts’s law does not hold^22^. In this task, the participants had to support the weight of their arm by themselves and mostly moved in a transverse plane with a fully extended arm where the kinematics of leftward and rightward movements is known to be similar^48^ (in particular, velocity profiles are known to be symmetrical and bell-shaped and hand path is necessarily an arc of a circle).

Questionnaires: After the motor task, participants fulfilled two questionnaires that are commonly used to measure the personality traits of impulsiveness and boredom proneness. These questionnaires are the Barratt Impulsiveness Scale (BIS-10^49^) and the Boredom Proneness Scale (BPS^50^). The BIS-10 consists of 34 Likert-scale items (4-point) such as “I do things without thinking” or “I act on impulse” and the BPS includes 28 true-false items such as “Time always seems to be passing slowly” or “I am good at waiting patiently”. High scores in these scales suggest a psychological profile of impulsiveness and boredom proneness.

#### Data Collection and Processing

Materials: Motion of the head and of the dominant arm was recorded by means of a motion capture system (Optitrack device). Ten cameras were used to capture the movement of five retro reflective markers (15 mm in diameter) at a sampling frequency of 250 Hz. Markers were placed at well-defined anatomical locations on the moving arm and head, namely the acromial process, the humeral lateral condyle, the apex of the index finger, and the left and right sides of the frontal bone.

Data analysis: All the analyses were performed with custom software written in Matlab (Mathworks, Natick, MA) from the recorded 3D positions of markers. Recorded signals were low-pass filtered using a digital fifth-order Butterworth filter at a cutoff frequency of 10 Hz (Matlab butter/filtfilt functions). Cartesian velocity profiles were computed via numerical differentiation. Movement duration was determined by fitting experimental velocity profiles to minimum jerk velocity profiles^51^ as they are known to capture very well the bell-shaped nature of speed profiles in such planar arm reaching movements. The jerk velocity profiles depended on 3 parameters controlling the peak velocity (*a*), its onset and final times (*t*_0_ and *t*_*f*_), and were expressed as follows (outside the interval [*t*_0_, *t*_*f*_] velocity was set to zero):

4 $${v}_{a,{t}_{0},{t}_{f}}(t)=a[\frac{\mathrm{60(}t-{t}_{0}{)}^{3}}{{({t}_{f}-{t}_{0})}^{4}}-\frac{\mathrm{30(}t-{t}_{0}{)}^{4}}{{({t}_{f}-{t}_{0})}^{5}}-\frac{\mathrm{30(}t-{t}_{0}{)}^{2}}{{({t}_{f}-{t}_{0})}^{3}}].$$

To do so, a global optimization using Matlab’s *patternsearch* function was carried out and the parameters of the best-fitting minimum jerk problem were identified, i.e. movement start, end and its total duration (see^52^ for a similar approach). For example, determination coefficients between the best-fitting minimum jerk speed profiles and the empirical ones were *R*^2^ = 0.98 ± 0.07 for the illustrated participant. The shoulder angle was eventually estimated from the shoulder and finger markers using the inverse trigonometric function of cosine (*acos* of the dot product between unit vectors defining the initial and end arm orientation). Movement amplitude was then computed as the difference between the estimated final angular position and starting one (in degrees).

#### Definition of movement vigour

To define movement vigour, we started from the experimental observation that duration *T* approximately increased in an affine way as a function of amplitude *A*, for these self-paced arm pointing movements, i.e. *T* = *aA* + *b* with *a*, *b* > 0 (see Results for quantitative data). This was the simplest possible fit to the data to characterize the overall behaviour of a participant in the present task. Other fits were tested, especially according to the formula given in^22^, i.e. $T=aA+b+c\,{log}_{2}(A/W+\mathrm{1)}$ where *W* is the width of the target (about 2 degrees here on average). Slight fitting improvements were obtained (*R*^2^ = 0.99 ± 0.03) but with one additional parameter. These fits imply that mean velocity was of the form $V=\frac{A}{aA+b}$ or $V=\frac{A}{aA+b+c\,{\mathrm{log}}_{2}(A\,/\,W+1)}$ such that for large amplitudes $(A\gg 1)$, *V* would attain an asymptotic value equal to 1/*a*. This saturation is similar to what is observed for saccades and walking, and it makes sense biologically as it is more likely that velocity, rather than duration, will saturate in the limit of very large distances. Vigour was then simply defined as the inverse of the slope of this affine amplitude-duration relationship. A relative value of vigour was obtained by normalizing the vigour scores with respect to the mean score measured across all participants. A similar relative vigour score was defined for saccades in^6^, based on the relationship between amplitude and peak velocity which exhibits a similar graph passing through the origin. For the sake of comparison, we also computed vigour scores exactly as in^6^ (without resorting to any fitting) and found positive correlation between our vigour scores based on 1/*a* and their vigour scores, across participants (R = 0.96). The intercept *b* may also capture another aspect of movement vigour. In this study, we however decided to focus on how an individual increases duration with amplitude and assumed it better represented the notion of vigour. Hence, an individual may move faster than another individual with the same slope if intercept is greater but our definition would imply that these two individuals have the same vigour. Therefore, we define vigour up to an offset value related to intercepts. In practice, however, slopes (*a*) and intercepts (*b*) data tend to correlate (see Results). Our modeling nevertheless takes into account both slope and intercept since the actual movement times of each individual will be used to infer time costs.

### Modeling of the effort/time compromise within optimal control theory

In^4,30^, we showed how the CoT can be recovered from empirical motion data, under certain assumptions. Briefly, this is done within the optimal control framework by formulating the motor planning problem in free time (duration then becomes an emergent property of optimality). For the single-joint arm moving in the horizontal plane considered in the present study, the joint torque was computed $\tau =I\ddot{\theta }+b\dot{\theta }$ where *θ* is the shoulder joint angle, *τ* is the net joint torque coming from the net action of muscle forces, *b* is the friction coefficient (fixed and set to 0.8), *I* is the moment of inertia of the arm with respect to the shoulder (value estimated based upon Winter’s table for each participant using an estimation of the radius of gyration and the mass of the whole arm^27^). The scalar control variable was *u* = $\dddot{{\theta }}$, that is, the angle jerk. We did not model muscle dynamics here because the theory can be derived at a high-level of the motor hierarchy^2^ and modeling low-level dynamics such as the muscle excitation/activation processes would introduce additional parameters that are hardly adjustable on an individual basis. The system state for this dynamical system was written $x={({\theta },\dot{{\theta }},\ddot{{\theta }})}^{\top }$. The optimal control model we considered was linear-quadratic, as follows:

5 $$\dot{{\bf{x}}}=A{\bf{x}}+Bu\,{\rm{and}}\,C(T)={\int }_{0}^{T}[u{(t)}^{\top }Ru(t)+{\bf{x}}{(t)}^{\top }Q{\bf{x}}(t)]dt+{\int }_{0}^{T}g(t)dt$$

with

6 $$A=(\begin{array}{ccc}0 & 1 & 0\\ 0 & 0 & 1\\ 0 & 0 & 0\end{array}),\,B=(\begin{array}{c}0\\ 0\\ 1\end{array}),\,Q=(\begin{array}{ccc}0 & 0 & 0\\ 0 & {b}^{2} & Ib\\ 0 & Ib & {I}^{2}\end{array}),\,{\rm{and}}\,R=\varepsilon .$$

In this formulation, $L(T)={\int }_{0}^{T}[{u}^{\top }Ru+{{\bf{x}}}^{\top }Q{\bf{x}}]dt$ is a measure of the biomechanical cost of movement, which depends on the control variable *u*(*t*) and on the trajectory ***x***(*t*). More importantly for our purpose, $G(T)={\int }_{0}^{T}g(t)dt$ is the “cost of time” and movement time *T* is left free. Note that we chose *G*(0) = 0 for simplicity and that the origin of time is taken at movement onset in this formulation. A mathematical analysis shows that it is possible to compute the value *g*(*T*) by solving the associated optimal control problem in fixed time *T* which only minimizes the effort cost *L*(*T*). Movement duration *T* and the initial/final states of the system can be taken from experimental data or fits of the experimental data to reduce the effect of sensorimotor noise (which makes sense since we reason at the motor planning level). Once this is done, we can compute the value *g*(*T*) as the partial time derivative of the *value* function of the associated fixed-time optimal control problem (i.e. without time cost) or, equivalently, as the value of the maximized Hamiltonian for that problem^53^. Details for the calculation of *g*(*T*) are provided as Supplementary Information text where an example of a Matlab code is also provided. Note that the total cost *C*(*T*) is defined up to a positive multiplicative factor in this formulation. If one takes *λL*(*T*) in place of *L*(*T*) as a measure of effort for any *λ* > 0, one would identify *λG*(*T*) in place of *G*(*T*), but all other predictions would remain the same (same optimal movement times, joint trajectories etc.). For this reason, we decided not to implement any normalization and to consider the biomechanical cost of movement as it is.

In practice, the above methodology allows sampling a value *g*(*T*) from each movement time *T*. In self-paced reaching movements, different movement times can be obtained by varying the instructed motion amplitude as we did. Therefore, we can sample *g*(*T*) values for times *T* ranging from the shortest to the longest recorded movements, an interval that depends on each participant. Under these assumptions (precisely, affine or log-affine fits of the amplitude-duration data, quadratic cost of movement based on torque or torque change, see Supplementary Information), the shape of *g* revealed that *G* should be a sigmoidal function (by visual inspection, we noticed that the sigmoid could even be asymmetric, see Supplementary Information). Hence, we decided *a posteriori* to fit the estimated time costs *G* according to the sigmoidal function defined in Equation 3. In practice, since we sampled *g*, we fitted the following function in order to identify the three best-fitting parameters (using a nonlinear least squares method):

7 $$g(t)=\frac{\alpha \beta }{\delta }\frac{{(\frac{t}{\delta })}^{\beta -1}}{{(1+{(\frac{t}{\delta })}^{\beta })}^{2}}.$$

### Statistical decomposition of variance

Hierarchical linear models (HLM^54^) were used to obtain a statistical decomposition of inter-individual versus intra-individual variances for vigour scores. We provide below a brief outline of this technique. HLM allows for the testing of multilevel data with a hierarchically nested structure (e.g., a set of subjects, each tested in different conditions such as blocks or sessions). This model has several advantages over traditional methods such as repeated-measures ANOVA that only give a descriptive account of the decomposition of inter-group vs intra-group variance via the partitioning of the sum of squares. If each group refers to a block or session, intra-group variability would give inter-individual variance whereas inter-group variance would lead to inter-block or inter-session variances. As such, it cannot capture intra-individual variability. If each group is a single participant, ANOVA will just provide a descriptive decomposition of intra- versus inter-individual variance. We therefore considered a multilevel modeling approach.

A common way to conceptualize a multilevel model in the present experiment is through three levels of analysis^55^. Levels 1, 2 and 3 respectively refer to inter-block, inter-session and inter-individual variabilities. Levels 1 and 2 thus capture intra-individual variability. Precisely, if we denote the block id by *i*, the session id by *j* and the subject id by *k*, the model tested here (called empty model or model 1 in this work) writes:

8 $${y}_{ijk}={\beta }_{0}+{u}_{k}+{v}_{jk}+{e}_{ijk}$$

where *y*_*ijk*_ is the vigour score and *β*_0_ is its grand mean. At the third level, *u*_*k*_ is a random term characterizing the deviation of each individual’s mean value from *β*_0_. At the second level, *v*_*jk*_ is the random term describing the deviation between sessions with respect to the individual mean. Finally, at the first level, *e*_*ijk*_ is the residual term describing the deviations between blocks from the mean value for each session and individual. Assuming that ${e}_{ijk} \sim N(\mathrm{0,}\,{{\rm{\Omega }}}_{e})$, ${v}_{jk} \sim N\mathrm{(0,}\,{{\rm{\Omega }}}_{v})$ and ${u}_{k} \sim N\mathrm{(0,}\,{{\rm{\Omega }}}_{u})$ we can now estimate their variances and their significance at level 1 (Ω_*e*_), level 2 (Ω_*v*_) and level 3 (Ω_*u*_). This allows to reliably identify at which level (1, 2 or 3) the total variance in vigour is mainly rooted and conclude about intra-individual variability relative to inter-individual variability, for the motor task under consideration.

### Data availability

The raw data that support the findings of this study are available from the corresponding author upon request.

### Code availability

The code used for the simulations in this study is available from the corresponding author upon request.

## Electronic supplementary material

Supplementary Information
