# Supplementary material for: Vigour of self-paced reaching movement: cost of time and individual traits
Source: Sci Rep. 2018 Jul 13;8:10655. doi: 10.1038/s41598-018-28979-6 (PMC6045586; doi:10.1038/s41598-018-28979-6)
Supplement: Supplementary file 1 — Supplementary Information [file 41598_2018_28979_MOESM1_ESM.pdf]

Vigour of self-paced reaching movement: cost of time and individual traits

Bastien Berret, Carole Castanier, Simon Bastide and Thomas Deroche

This supplementary text gives details about how to compute the cost of time (CoT) value $g(T)$ for a given time T. It also provides complementary analyses to test the sensitivity of the results to different modeling choices.

Identification procedure of the CoT value $g(T)$

To calculate the value $g(T)$, for a given time T, we consider the fixed-time optimal control problem (in time T) associated with the free-time problem formulated in the main text (which includes the time cost g). We recall that the dynamical system under consideration is linear as follows:

$$\dot{\mathbf{x}} = A\mathbf{x} + Bu$$

where $\mathbf{x} = (\theta, \dot{\theta}, \ddot{\theta})^\top$ is the system state and u the control variable.

The goal is to find the optimal control $u(\cdot)$ and the associated trajectory $\mathbf{x}(\cdot)$ joining an initial state $\mathbf{x}(0) = \mathbf{x}_0$ to a final state $\mathbf{x}(T) = \mathbf{x}_f$ that minimize the following integral cost (called physical effort):

$$C(u) = \int_0^T [u^\top R u + \mathbf{x}^\top Q \mathbf{x}]\, dt.$$

To solve this problem, we use Pontryagin Maximum Principle [1] which gives necessary optimality conditions, and define the Hamiltonian as follows:

$$\mathcal{H}(u, \mathbf{x}, \mathbf{p}) = u^\top R u + \mathbf{x}^\top Q \mathbf{x} + \mathbf{p}^\top (A\mathbf{x} + Bu)$$

where $\mathbf{p}$ is the co-state (or adjoint) vector.

We can compute the optimal control that minimizes the Hamiltonian with respect to u and get:

$$u^\star = -\frac{1}{2} R^{-1} B^\top \mathbf{p}.$$

A star denotes a quantity related to the optimal solution. The co-state equation is given by:

$$\dot{\mathbf{p}} = -\frac{\partial \mathcal{H}}{\partial \mathbf{x}} = -A^\top \mathbf{p} - Q\mathbf{x}$$

and thus:

$$\dot{\mathbf{x}} = A\mathbf{x} - \frac{1}{2}BR^{-1}B^\top \mathbf{p}$$

Furthermore, we know that for this time-invariant problem the minimized Hamiltonian $\mathcal{H}^\star$ will be constant along the optimal trajectory:

$$\mathcal{H}^\star = -\frac{1}{4}\mathbf{p}^\top BR^{-1}B^\top \mathbf{p} + \mathbf{x}^\top Q\mathbf{x} + \mathbf{p}^\top A\mathbf{x} = cst$$

Let us define the matrix:

$$H = \begin{pmatrix} A & -\frac{1}{2}BR^{-1}B^\top \\ -Q & -A^\top \end{pmatrix}$$

Defining $\xi = \begin{pmatrix} \mathbf{x} \\ \mathbf{p} \end{pmatrix}$, we get the hamiltonian system

$$\dot{\xi} = H\xi$$

whose solution writes as follows:

$$\xi(t) = \Phi(t)\xi_0$$

where the state-transition matrix is $\Phi(t) = \exp(Ht)$ and $\xi_0 = \begin{pmatrix} \mathbf{x}_0 \\ \mathbf{p}_0 \end{pmatrix}$.

We can partition the matrix $\Phi(t)$ in blocks as follows:

$$\Phi(t) = \begin{pmatrix} \Phi_{11}(t) & \Phi_{12}(t) \\ \Phi_{21}(t) & \Phi_{22}(t) \end{pmatrix}.$$

From this block matrix and $\xi(t) = \Phi(t)\xi_0$, we conclude in particular that:

$$\mathbf{x}_f = \Phi_{11}(T)\mathbf{x}_0 + \Phi_{12}(T)\mathbf{p}_0 = \mathbf{0}.$$

Therefore, if $\Phi_{12}^{-1}(T)$ exists (which is the case if the system is fully controllable), we get:

$$\mathbf{p}_0 = \Phi_{12}^{-1}(T)\Phi_{11}(T)\mathbf{x}_0.$$

Since the Hamiltonian is constant along the optimal trajectory we obtain its value as follows:

$$\mathcal{H}^\star = -\frac{1}{4}\mathbf{p}_0^\top BR^{-1}B^\top \mathbf{p}_0 + \mathbf{p}_0^\top A\mathbf{x}_0.$$

Finally, the infinitesimal CoT at time T is (see [2, 3]):

$$g(T) = -\mathcal{H}^\star = \frac{1}{4}\mathbf{p}_0^\top BR^{-1}B^\top \mathbf{p}_0 - \mathbf{p}_0^\top A\mathbf{x}_0.$$

Note that only one matrix exponential, i.e. $\exp(HT)$, needs to be computed in order to obtain the value $g(T)$. If we repeat this procedure for different durations T we can get different values $g(T)$ and,

therefore, we can infer the shape of $g(\cdot)$ on some time interval, and eventually integrate it to recover the genuine cost of time $G(T) - G(0) = \int_0^T g(t)dt$.

Example of Matlab code

```
% Example of script to compute g(T) values
amps=linspace(5,95,20)*pi/180; % amplitudes of stimuli
durs=0.008*amps*180/pi+0.64; % corresponding recorded durations
% anthropometric data (to adjust for each subject)
I=0.45; % moment of inertia
b=0.8; % friction coefficient
% system dynamics : xdot=Ax+Bu
% x=[angular position, angular velocity, angular acceleration]
% u=angular jerk
A =[0 1 0; 0 0 1; 0 0 0];
B = [0; 0; 1];
ns=size(A,2); % dimension of state vector
% quadratic cost int_0^T [u'Ru + x'Qx] dt
R=0.005;
invR=1/R;
Q=[0 0 0; 0 b^2 I*b; 0 I*b I^2];
for i=1:numel(durs)
        % experimental motion data
        a=amps(i); % motion amplitude
        T=durs(i); % corresponding motion duration
        x0=[a,0,0]'; % initial state
        xf=[0,0,0]'; % final state
        % transition matrix at final time T
        PhiT=expm([A,-(1/2)*(B*invR*B'); -Q, -A']*T);
        % initial co-state vector
        p0=PhiT(1:ns,ns+1:end)\(PhiT(1:ns,1:ns)*x0);
        % Infinitesimal value g(T)
        gT=(1/4)*p0'*(B*invR*B')*p0-p0'*A*x0;
        g(i)=gT; % inferred g(T) value
end
% plot infinitesimal cost of time g(T)
plot(durs,g,'.-'); xlabel('Time'); ylabel('CoT g(T)');
```

Link between CoT parameters and traits for asymmetric sigmoids

In the main text, we fitted the CoT to symmetrical sigmoid functions. Here we fitted the CoT to asymmetric sigmoids of the form $G(T) = \alpha - \alpha/(1+(\frac{T}{\delta})^{\beta})^{0.1}$. Indeed, visual inspection indicated that the inferred time costs may be asymmetrical logistic functions.

Fits were more accurate than when using standard symmetric sigmoids ($R^2 = 0.96 \pm 0.02$).

Here are the regression analyses (Fig. 1).

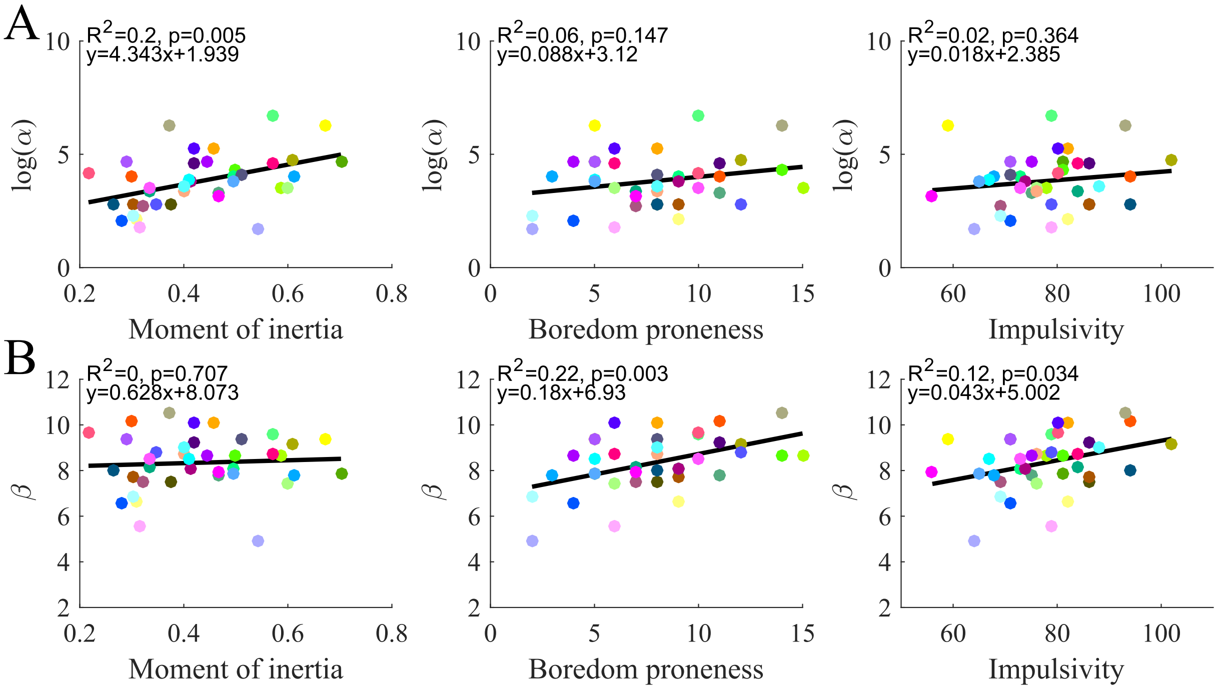

Figure 1: *Regression analyses between the main CoT parameters and individual traits.* A. Relationships between moment of inertia, boredom proneness, impulsivity, and $\log\alpha$. B. Relationships between moment of inertia, boredom proneness, impulsivity, and β.

Link between CoT parameters and traits for the minimum torque change cost

Here we tested a different measure of the biomechanical cost of movement. We considered a cost based on the minimum torque change model. We fitted time costs to standard sigmoids. Goodness of fit was $R^2 = 0.88 \pm 0.04$.

The results of the regression analyses are presented in Figure 2.

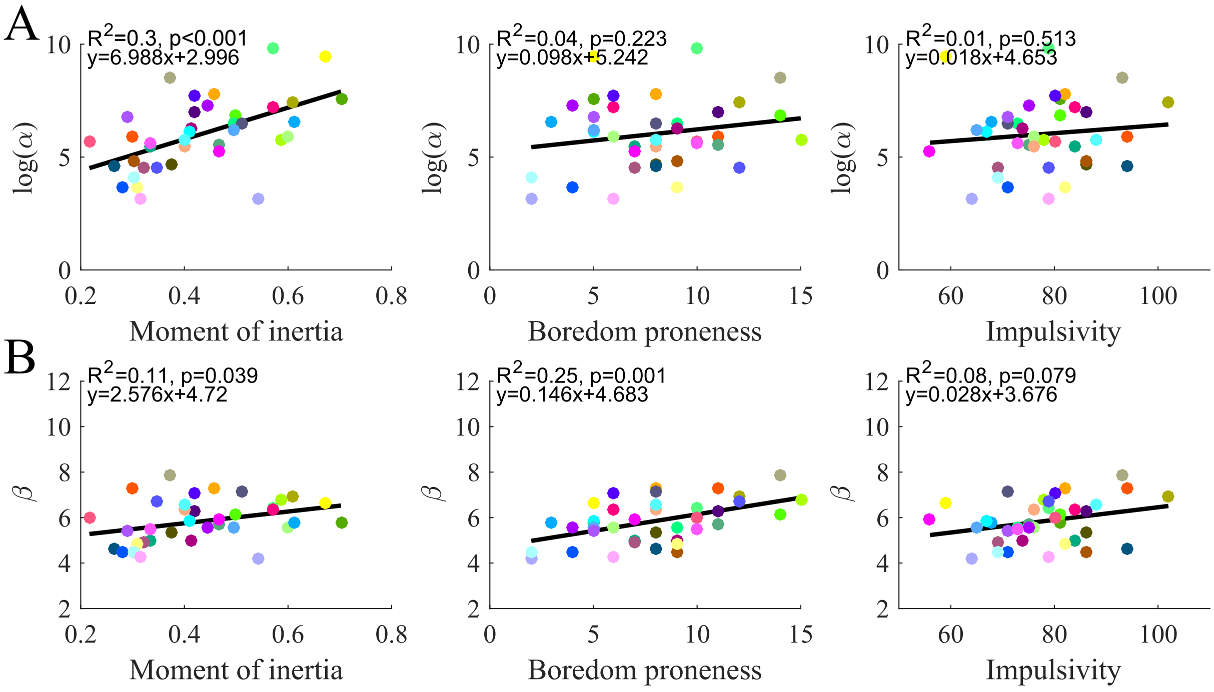

Figure 2: *Regression analyses between the main CoT parameters and individual traits.* A. Relationships between moment of inertia, boredom proneness, impulsivity, and $\log \alpha$. B. Relationships between moment of inertia, boredom proneness, impulsivity, and β.

References

1. Pontryagin, L. S., Boltyanskii, V. G., Gamkrelidze, R. V. & Mishchenko, E. F. *The Mathematical Theory of Optimal Processes.* (Pergamon Press, 1964).

2. Berret, B. & Jean, F. Why don't we move slower? the value of time in the neural control of action. *J Neurosci* **36**, 1056–1070 (2016).

3. Jean, F. & Berret, B. On the duration of human movement: from self-paced to slow/fast reaches up to fitts's law. In *Geometric and Numerical Foundations of Movements*, 43–65 (Springer, 2017).
